# The impact of the opening of high-speed rail on corporate financing constraints

**DOI:** 10.1371/journal.pone.0268994

**Published:** 2022-06-06

**Authors:** Yu Liao, Xiaodong Qiu, Qian Sun, Peiyang Li

**Affiliations:** 1 School of Economics and Management, Beijing Jiaotong University, Beijing, China; 2 Research Center for Central and Eastern Europe, Beijing Jiaotong University, Beijing, China; 3 School of Economics and Management, Nanjing University of Science and Technology, Nanjing, China; The Bucharest University of Economic Studies, ROMANIA

## Abstract

In this paper, the data of A-share non-financial listed companies from 2008 to 2019 are used to study the impact of the opening of high-speed railway on corporate financing constraints with the difference-in-differences model. The research results show that the opening of high-speed rail can effectively alleviate the financing constraints faced by enterprises. Through the analysis of its influence mechanism, it is found that the opening of high-speed rail can reduce the degree of information asymmetry and agency conflicts between enterprises and their stakeholders, and thereby ease corporate financing constraints. At the same time, the opening of the high-speed rail has a more significant effect on alleviating the financing constraints of non-state-controlled enterprises, technology-intensive enterprises, and enterprises in inland areas. This research is not only a supplement to the economic consequences related to the opening of the high-speed rail, but also a further expansion of the research on the factors affecting corporate financing constraints.

## 1. Introduction

Since the opening of the Beijing-Tianjin Intercity Railway in 2008, Chinese high-speed rail construction has continued to make breakthroughs and innovations. After more than ten years of development, China’s high-speed rail has become the best in the world in terms of operating speed, operating mileage, transportation density, and high-speed rail traffic. As of the end of 2020, China’s high-speed rail operating mileage has reached 38,000 kilometers, ranking the first in the world.

The opening of the high-speed rail has significantly promoted the rapid development of China’s economy. Business operations, cultural interoperability, and economic structure have all been affected to varying degrees by the opening of high-speed rail. Studies have found that the opening of high-speed rail directly or indirectly affected regional employment, wages, and economic growth space, reshaped China’s economic space [1], significantly improved the spillover effect of China’s inter-regional economic growth and regional innovation level [2, 3]. In terms of micro-enterprises, the opening of the high-speed rail helps to improve the suppression of private equity investment by geographical location, better exerts the governance effect on enterprises and the market [4], improves the local information environment and expand the credit scale of locally listed companies [5], alleviates the information asymmetry between enterprises along the high-speed rail line and external investors, realizes the optimal allocation of entrepreneurial market and capital market resources [6–8], and promotes enterprise innovation [9]. At present, the research on the economic consequences of the opening of the high-speed rail still focuses on the regional economy and other macro-level research, while researches on the micro level of enterprises mostly focus on enterprise investment, internal management and other aspects. The influence on enterprise financing needs to be further discussed. The MM theory proposed by Modigliani and Miller (1958) believes that in a perfect capital market, companies can raise funds for valuable investment projects in the external capital market at the same cost of capital as internal, and the investment behavior of enterprises will not be affected by their own financial status, but only related to the investment needs of enterprises [10]. However, in the actual capital market, there are still many imperfections in the capital market system. Due to the existence of information asymmetry and agency problems between management and investors, the company’s investment decisions will be affected by the availability of internal and external funds. As a result, some investment opportunities with a positive net present value are forced to abandon, making investment decisions unable to reach the optimal level, which leads to financing constraints.

Will the opening of high-speed rail affect the financing constraints of enterprises? If so, by what means? In response to the above problems, this paper selects 2008–2019 A-share non-financial listed companies as sample data. Based on the quasi-natural experiment of high-speed railway opening, this paper discusses the influence of high-speed railway opening on financing constraints of enterprises and its mechanism by using the difference-in-differences method. The results show that after the opening of the high-speed rail in the office of listed companies, the degree of corporate financing constraints has been significantly eased. Through a more in-depth analysis of its mechanism, it is found that the opening of the high-speed rail greatly reduces the space-time distance and improves the accessibility of cities along the route, thereby reducing the degree of information asymmetry, alleviating the agency conflicts of the companies, and thereby alleviating the financing constraints of the enterprise.

The possible contributions and innovations of this paper are as follows: (1) With the help of the exogenous event of the opening of high-speed rail, exploring the impact of transportation infrastructure conditions on the company’s financing constraints will help provide a new perspective for studying the factors affecting company financing constraints. (2) Existing literature researches on the micro-level of enterprises with the opening of high-speed railway mostly focus on the internal management efficiency and investment efficiency of enterprises. This paper takes corporate financing constraints as the foothold and conducts related research from the perspective of corporate financing. It will help to provide the new empirical evidence for the economic consequences of the opening of the high-speed rail from the micro-level, and enrich the literature on the consequences of the opening of the high-speed rail. (3) Under the theoretical framework of new geographic economics, this paper takes the opening of the high-speed railway as an exogenous event to explore the impact of the opening of high-speed railway on the financing constraints of enterprises, which is helpful to expand the application scope of new geographic economics in the fields of corporate finance and corporate governance.

The rest of the paper is based on the following framework: The second part builds a theoretical analysis framework and proposes research hypotheses, the third part is the research design content, the fourth part is the empirical research results and analysis of the article, and the fifth part is the analysis of the influencing mechanism, the sixth part is the conclusion and policy recommendations of this article.

## 2. Theoretical analysis and research hypothesis

In recent years, the construction of transportation infrastructure in China has developed rapidly. The opening of high-speed rail has greatly reduced the space-time distance, shortened the distance between economic entities, improved the mobility between people and information, reduced the cost of information acquisition, and improved the efficiency of information dissemination. On the one hand, the opening of the high-speed rail reduces the travel and time costs of all parties, helps investors and analysts to conduct on-site investigations on the company more efficiently and frequently [11], and reduces the cost of acquiring "soft information" and alleviates the degree of information asymmetry between investors and enterprises. On the other hand, the shortening of time and space distance can enhance the auditor’s supervisory ability. External auditors can effectively evaluate the self-interested behavior of corporate management through face-to-face conversations with internal employees, senior management, and other stakeholders [12]. The opening of high-speed rail strengthens the connection between enterprises and their stakeholders, reduces supervision costs, and thus can effectively reduce the agency costs of enterprises. In addition, the opening of high-speed railway can also strengthen the supervision of stakeholders on the management of enterprises by reducing the cost of information acquisition and alleviate the agency problems of enterprises.

Modigliani and Miller (1958) believed that in a perfect capital market, the internal and external capital of a company can replace each other, and the investment behavior of a company is only related to the company’s own investment needs, and will not be affected by the company’s financial status at all. However, in the actual capital market, due to the existence of factors such as information asymmetry and agency problems, the external financing cost of a company is usually higher than the internal financing cost, which triggers financing constraints for the company. Existing research has confirmed that the higher the degree of information asymmetry of the enterprise, the higher the premium will be demanded by investors, and the higher the financing cost of the enterprise [13]. The higher the degree of information asymmetry between the company and investors, the greater the difference between the company’s internal financing cost and external financing cost, and the more serious the financing constraint of the company [14]. At the same time, in the case of information asymmetry, the external investors are the principal, managers as entrusted to maximize their own interests are likely to infringe on the rights and interests of investors, and outside investors can only demand a certain premium from the users of funds to guard against potential risks. To some extent, this also leads to the external cost of capital is higher than the internal cost of capital. Agency problems are also the cause of corporate financing constraints [15, 16].

Information asymmetry theory believes that in a market economy, there are differences in the understanding of information between parties, which will lead to problems such as unfairness in transaction relationships or low market efficiency. In the capital market, if fund owners and demanders cannot understand each other, it will affect the size of loans and market interest rates [17]. When the scale of loans is restricted, the demand for funds by the demander of funds is greater than the amount that the bank can provide credit allocation problems will arise [18, 19]. When there is asymmetric information between external investors and enterprises, external investors will require the use of external funds to pay premiums, which will trigger an increase in market interest rates and further increase financing costs [13]. From the perspective of the company’s cash holdings, the higher the degree of information asymmetry faced by the enterprise, the more cash it reserves, the greater its cash-cash flow sensitivity, and the greater the degree of financing constraints [20]. However, no matter through public information disclosure channels, or telephone or video communication, it is more effective to visit the company to obtain private information [21, 22]. Quantifiable "hard information" such as financial indicators can be obtained through public information collection and telephone communication. However, it is difficult to obtain "soft information" such as corporate culture, human capital and operating environment [23]. Field visits are the primary way to obtain corporate soft information [7, 24]. The efficiency of field visits mainly depends on the accessibility of transport infrastructure. To a certain extent, the opening of the high-speed railway provides convenient conditions for information users to further understand enterprise soft information and alleviate information asymmetry [25]. The opening of the high-speed rail breaks the barriers between cities, making it easier for both companies and investors to conduct on-site investigations and face-to-face communication, reducing the cost of information acquisition, improving the timeliness and symmetry of information acquisition by both parties, and effectively alleviating problems such as inconsistent sources of information acquisition, lag of information acquisition time, information acquisition asymmetry and so on, thus easing the degree of financing constraints of enterprises.

Agency theory believes that when the company’s ownership and control rights are inconsistent, to maximize their interests, operators will infringe on the shareholders’ rights and interests [26]. this raises the agency problem. In the process of the separation of ownership and control, the conflict between the maximization of shareholders’ interests and the management’s personal interests gradually becomes apparent, leading to the emergence of the first type of agency problem. With the increase of the concentration of controlling shareholders’ equity, the interests of controlling shareholders and minority shareholders diverge and conflict, leading to the second type of agency problem. At present, there are two kinds of agency problems in most listed companies. To ensure their interests, investors and creditors usually require companies to use external financing funds to pay a premium to compensate for the agency costs incurred, which leads to increased external capital costs [15, 16]. The significant increase in the cost of shareholder supervision and the difficulty of supervision caused by geographical distance has led to a low degree of supervision by shareholders, which is convenient for management to seize private interests, and the company’s agency problem is more prominent [27]. A distant corporate location increases the cost for shareholders to monitor the management of corporate investment decisions, thereby exacerbating agency conflict problems [28]. The opening of the high-speed rail makes it easier for corporate stakeholders to supervise corporate operations and management activities, reduces the high transaction costs between companies and stakeholders due to the time and space distance, and effectively reduces stakeholders’ supervision costs for the company. The easing of agency problems alleviate the financing constraint degree of enterprises. Based on the above analysis, this paper proposes Hypothesis 1:

H1: The opening of high-speed rail can ease the financing constraints of enterprises.

Under the background of China’s special system, compared with non-state-owned enterprises, state-owned enterprises have obvious advantages in property rights protection, government-enterprise relationship, financing treatment, etc. [29]. The nature of property rights has a significant impact on financing constraints of enterprises [30]. Fluctuations in the macroeconomic environment and policies, and excessive government intervention will aggravate corporate financing constraints to a certain extent. During the economic recession, the profitability of enterprises will be impacted by the economic environment, showing a downward trend, and the uncertainty of the macroeconomic environment will lead creditors to increase the premium level to deal with risks, thus increasing the external financing cost of enterprises and aggravating the degree of financing constraints of enterprises [15, 16]. Compared with state-controlled enterprises, the degree of financing constraints of non-state-controlled enterprises is more sensitive to changes in the macro-environment [31]. The degree of financing constraints faced by state-owned enterprises is generally less than that of private enterprises [32]. At the same time, state-owned enterprises assume more social responsibilities in maintaining the national economy. When state-owned enterprises face financing difficulties, the government will lend a helping hand to them. Compared with non-state-owned enterprises, state-owned enterprises have more advantages in financing resources. State-owned enterprises have greater control over the financial resources they receive, while large non-state-owned enterprises lacking government support are more vulnerable to financial constraints [30]. Therefore, due to the strong support of the government, the change of transportation infrastructure will not have a significant impact on the financing constraints of State-Owned enterprises. This leads to the second hypothesis of this paper:

H2: When the property rights of enterprises are non-state-owned, the opening of high-speed rail will have a more significant effect on alleviating corporate financing constraints.

Different industries have different development directions and business strategies, and their financing methods, financing scale and financing constraints are also different. The opening of high-speed rail also has different alleviating effects on financing constraints of different industries. In their research, Lu Tong and Dang Yin (2014) classified the industries of all listed companies in China into labor-intensive, capital-intensive and technology-intensive according to factor intensity [33]. Labor-intensive enterprises mainly rely on individual workers, have little demand for capital and technology, and face relatively small degree of financing constraints. The opening of the high-speed railway has a relatively limited impact on the financing constraints of labor-intensive enterprises. Although capital-intensive enterprises there are a lot of money demand, enterprise operation requires a lot of money, but on the other hand, capital-intensive enterprises have high prestige and status in society, many factors such as support from the government, and the enterprise itself has the strong ability of financing, so the high-speed railway opening degree of impact on its financing constraints is relatively small. For technology-intensive enterprises, the pursuit of innovation is the main characteristic of this industry, which requires a large amount of financial support for its operation. However, due to the high degree of information asymmetry in this industry, investors face higher investment risks and enterprises face greater financing constraints. The difficulty of technological innovation increases with the narrowing of the technological gap. According to the new structural economics, industries with more comparative advantages may be more affected by the opening of the high-speed railway as factor allocation conforms to regional factor endowment [34]. The opening of the high-speed railway can reduce the degree of information asymmetry between enterprises and their stakeholders, accelerate the flow of personnel between cities, help technology-intensive enterprises attract more technical talents, improve the innovation vitality of enterprises, and further ease the degree of financing constraints of enterprises. Therefore, the third hypothesis of this paper is proposed:

H3: When enterprises are in technology-intensive industries, the opening of high-speed rail has a more significant effect on alleviating corporate financing constraints.

Financing constraints are not only affected by internal factors of an enterprise such as its size, growth and asset structure, but also by the level of economic and financial development of a country (region) [35]. As the geographical distance between listed companies and stakeholders increases, direct transaction costs such as circulation cost and travel cost of listed companies will rise, and a series of indirect costs such as information collection costs, communication costs and supervision costs will also increase. It can be seen that the geographical location of listed companies is closely related to their financing environment. China has a vast territory, and the differences in geographical conditions in different regions also lead to differences in the level of its economic development to a certain extent. Compared with inland areas, coastal areas have a higher degree of openness and marketization, and faster economic development, which can provide a relatively loose financing environment for enterprises. At the same time, the transportation infrastructure in coastal areas is more convenient, which also provides more convenient conditions for information exchange between enterprises and their stakeholders, which can alleviate the financing constraints of enterprises to a certain extent. For companies in inland areas, the degree of transportation convenience is relatively low, and the travel cost is relatively high, which is not conducive to fund providers to conduct on-the-spot research on the company, and it is not conducive for the company’s personnel to go out to actively seek financing channels, so the financing environment is relatively poor. The opening of high-speed railway can alleviate a series of indirect costs such as travel costs for both investment and financing parties, information communication and supervision, and improve the financing situation of enterprises. This leads to the fourth hypothesis of this paper:

H4: When the company’s office is located in the inland area, the opening of the high-speed rail has a more obvious effect on alleviating the financing constraints of the company.

## 3. Research design

### 3.1. Data selection

On August 1, 2008, China’s first high-speed railway line, the Beijing-Tianjin Intercity Railway, was officially opened to traffic. China has officially entered the era of high-speed rail. Therefore, this article selects all A-share non-financial listed companies from 2008 to 2019, referring to the relevant literature of Long Yu et al. (2017), we exclude the sample of municipalities directly under the Central Government and provincial and sub-provincial cities to ensure the exogenous effect of high-speed rail; exclude ST and *ST companies; and exclude samples with missing values. Finally get 11432 sample observations. To minimize the influence of extreme values on the conclusions of the study, all continuous variables are abbreviated at the 1% and 99% quantiles. The financial data in the sample are all from the CSMAR database, and the variables related to the opening of the high-speed rail are obtained from the official website of the China Railway Administration and Baidu searches. At the same time, to exclude the influence of other transportation facilities on the research conclusions, we manually collect the relevant data of flights, inland shipping, roads and railways from the website of the Civil Aviation Administration of China and the website of the National Bureau of Statistics of the People’s Republic of China.

### 3.2. Variable definition

#### 3.2.1. Explained variable

The explained variable in this paper is the degree of financing constraints faced by the companies. At present, there is no unified standard and method for the measurement of financing constraints. Referring to relevant literatures, its measurement method involves single company characteristic index method (company size, dividend payout rate, leverage ratio, etc.), but these single company characteristic index method fails to take into account the influence of accidental factors or abnormal circumstances, and therefore cannot accurately reflect the degree of financing constraints of a company. Lamont used the method of Kaplan and Zingales to measure the financing constraints of different enterprises and constructed KZ index [14, 36]. Along the same lines, Whited and Wu constructed the WW index based on quarterly financial statement data [37]. Hadlock and Pierce extended the method proposed by Kaplan and Zingales [38]. Firstly, according to the financial status of each enterprise, the enterprises are qualitatively divided into five types of financing constraints, and then uses the Ordered Probit model to estimate the SA index to measure the financing constraints of enterprises level. Many scholars have continued to use these indices as the measure of financing constraints in subsequent studies [39–41]. In order to eliminate the influence of index selection on the measurement of financing constraints, this paper uses three comprehensive index measurement methods represented by KZ index, WW index and SA index to measure the degree of financing constraints of the company. Meanwhile, the cash-cash flow sensitivity model [20] and investment-cash flow sensitivity model [42] are further used in the robustness test. The calculation methods of comprehensive indicators are as follows:

KZ=−1.0019CF+3.1392TLTD−39.3678TDIV−1.3147CASH+0.2826Q (1)

WW=0.938−0.091CF−0.062DIVPOS+0.021TLTD−0.044LNTA+0.102ISG−0.0335SG (2)

SA=−0.737Size+0.043Size*Size−0.04Age (3)

CF is the ratio of net cash flow from operating activities to total book assets; TLTD is the ratio of long-term interest-bearing liabilities to book assets; TDIV is the ratio of cash dividends to book assets; CASH is the ratio of monetary capital holdings to book assets; Q is Tobin’s Q value; DIVPOS is a dummy variable, and the value is 1 if the company pays dividends, otherwise it is 0; LNTA is the natural logarithm of the company’s total assets; ISG is the industry sales revenue growth rate; SG is the company’s actual sales revenue Growth rate; Size is the natural logarithm of the company’s total assets, indicating the company’s size; Age is the company’s establishment years. Since the calculated SA index is all negative, to facilitate comparison, this article refers to Ju X.S.’s practice to take the absolute value of the SA index [43]. The larger the calculated values of the three indicators, the greater the degree of financing constraints the company faces.

#### 3.2.2. Explanatory variables

The core explanatory variable of this paper is the dummy variable *(HSR*) of whether the high-speed rail is opened at the location of the company’s office. If the high-speed rail is opened in the t year of the company’s location, the HSR value is 1 in the current year and subsequent years, otherwise it is 0. At the same time, referring to relevant literature, the institutional investor shareholding ratio (*Institution*) is selected as the proxy variable of the degree of information asymmetry [44, 45], and the operating expense ratio (*Cost*) is selected as the proxy variable of the first type of agency problem [46, 47], and the difference between the control stake and the ownership stake of the ultimate controlling owner (Divergence) is selected as the proxy variable of the second type of agency problem [48].

#### 3.2.3. Control variables

Considering the influence of other factors on financing constraints, refer to the existing literature [49, 50], this article selects the size of the total net asset interest rate (*ROA*), and the debt-to-asset ratio (*LEV*), Whether the two roles are integrated (*Dual*), equity balance (*Balance*), working capital change (*ΔNWC*), fixed asset ratio (*PPE*), and board size (Board)as control variables. Considering the possible influence of other transportation modes on the conclusion of this paper, the annual passenger throughput (*InAir*), total railway mileage (*InRail*), total highway mileage (*InRoad*), and total inland river shipping mileage (*InWater*) of prefectural-level administrative regions where the company is located are also controlled in this paper. In this paper, firm and annual fixed effects are controlled and clustered at firm level. Specific variable definitions are shown in Table 1.

**Table 1:** Description of main variables.

Variable symbol	Variable definitions
*Constraint*	KZ index, WW index, SA index. Proxy Variables for Financing Constraints
*HSR*	If the high-speed rail is opened in the current year, the HSR value is 1, otherwise, it is 0
*Institution*	The proxy variable of the degree of information asymmetry, the proportion of listed company shares held by institutional investors
*Cost*	The agency variable of the first type of agency conflicts, (selling expenses + management expenses) / main business income
*Divergence*	The agency variable of the second type of agency conflicts, the difference between actual controller’s control and ownership
*SOE*	State-owned enterprises = 1, non-state-owned enterprises = 0
*Category*	Labor-intensive industries = 1, capital-intensive industries = 2, technology-intensive industries = 3
*Area*	Coastal areas = 1, inland areas = 0
*ROA*	Current net profit/total assets at the end of the period
*LEV*	Total liabilities/total assets at the end of the period
*Dual*	If the chairman and general manager are the same, the value is 1; otherwise, the value is 0
*Balance*	Number of shares held by no. 2–5 major shareholders/Number of shares held by the largest shareholder
*ΔNWC*	(Current working capital—Previous working capital)/Total assets at the beginning of the period
*PPE*	Net fixed assets/total initial assets
*Board*	The natural log of the number of directors
*InAir*	Natural logarithm of annual passenger throughput of prefecture-level administrative region where the company is located
*InRail*	The natural logarithm of the total railway mileage of the province in which the company is located
*InRoad*	The natural logarithm of total highway mileage in the province where the company is located
*InWater*	The natural logarithm of total inland waterway shipping mileage in the province where the company is located

### 3.3. Model

Construct a multi-period difference-in-differences model to test Hypothesis 1. The basic model is as follows:

$$Constraint_{i,t}=\alpha_{0}+\alpha_{1}HSR_{i,t}+\sum Controls+FirmEffects+YearEffects+\varepsilon_{i}$$ (4)

Among them: *Constraint*_*i*,*t*_ indicates the degree of financing constraint of enterprise i in year t (KZ index, WW index, SA index); *HSR*_*i*,*t*_ indicates whether the high-speed rail is opened in year t where enterprise i’s office is located, and the degree of impact of the opening of high-speed rail on corporate financing constraints is analyzed through its coefficient; *∑Controls* represents a series of control variables.

## 4. Empirical results and analysis

### 4.1. Descriptive statistics

**Fig 1:**
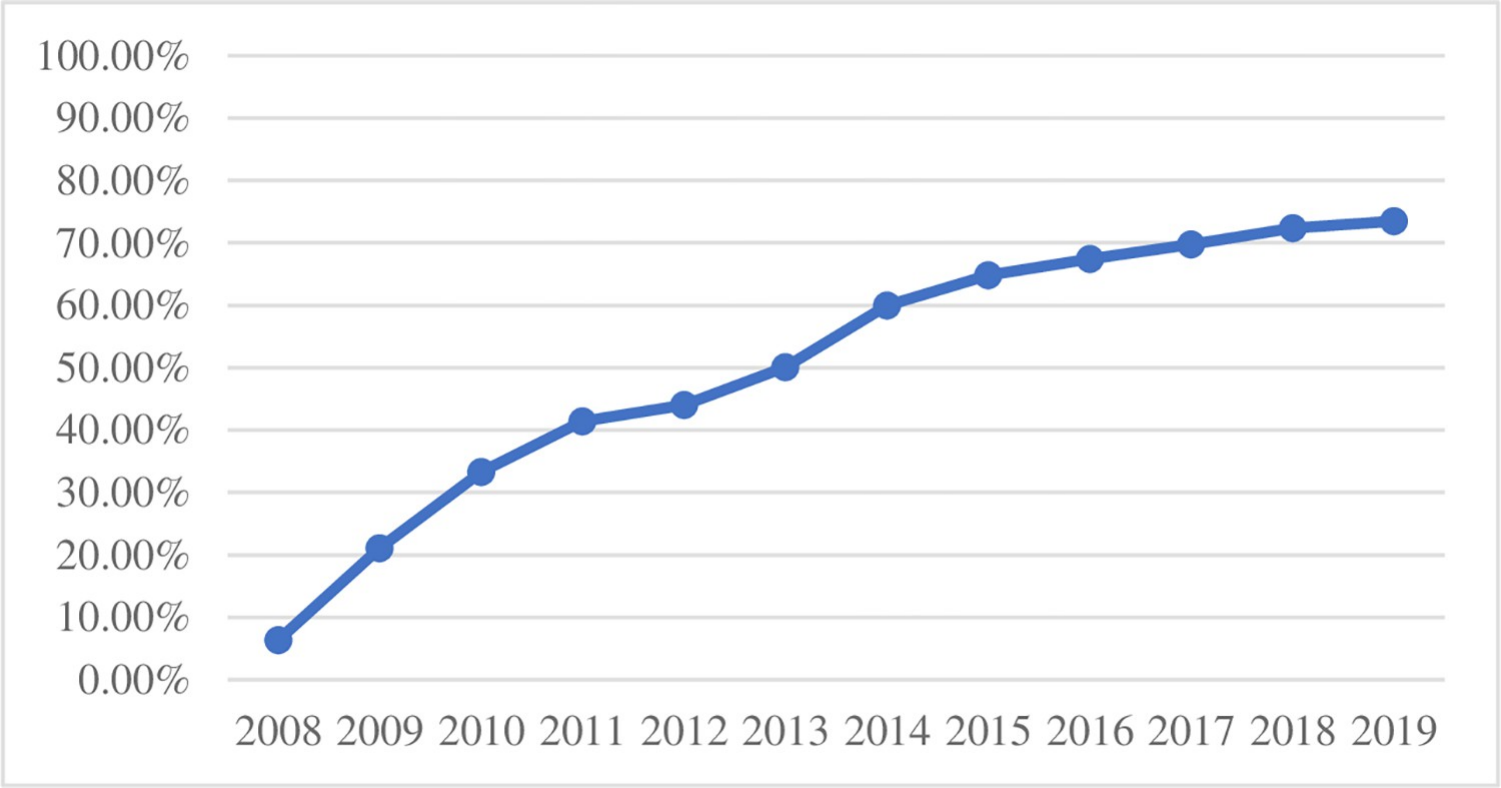
The proportion of companies that have opened high-speed rail in their locations.

**Fig 2:**
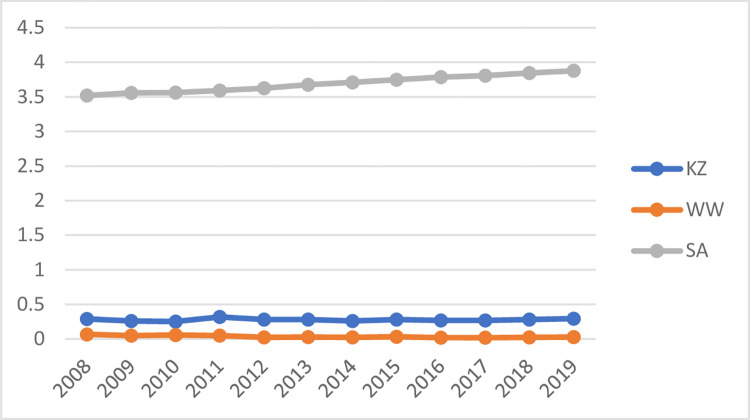
The trend of KZ\WW\SA index over time.

The descriptive statistical results of the main variables are shown in Table 2. The maximum values of KZ index, WW index and SA index are 3.863, 0.545 and 4.696, respectively. The minimum values are -1.011, -0.258, 2.818, respectively. It indicates that financing constraints exist widely in companies, and the degree of financing constraints varies greatly. The mean value of the explanatory variable HSR is 0.550, indicating that the sample of the treatment group accounted for 55.00% of the total sample. Therefore, it can be seen that the sample size of this paper is sufficient for research. Among the research samples, 29.30% of the samples are state-owned enterprises; 9.00% of the samples are located in the coastal areas of China, which is basically consistent with the overall distribution in China. Fig 1 describes the change of the proportion of enterprises with the high-speed railway in the sample with the passage of time. As can be seen from the chart, the proportion of enterprises with the high-speed railway in the area around the enterprise’s office increased from 6.35% in 2008 to 73.48% in 2019, showing an increasing trend year by year. Fig 2 depicts the changes in the sample mean of the three indices of KZ, WW, and SA over time. The results show that the financing constraints of enterprises are basically stable from 2008 to 2019, with no significant changes.

**Table 2:** Descriptive statistical results of main variables.

Variable	N	Mean	Std. Dev.	Min	Max
*KZ*	11,432	0.555	0.500	-1.011	3.863
*WW*	11,432	0.029	0.087	-0.258	0.545
*SA*	11,432	3.718	0.245	2.818	4.696
*HSR*	11,432	0.550	0.498	0.000	1.000
*Institution*	11,432	0.441	2.376	0.001	0.968
*Cost*	11,432	0.178	0.870	-0.001	0.875
*Divergence*	11,432	0.063	8.256	0.000	0.803
*SOE*	11,432	0.293	0.455	0.000	1.000
*Category*	11,432	2.175	0.802	1.000	3.000
*Area*	11,432	0.090	0.094	0.000	1.000
*ROA*	11,432	0.033	0.075	-0.382	0.202
*LEV*	11,432	0.443	0.213	0.056	0.979
*Dual*	11,432	0.270	0.444	0.000	1.000
*Balance*	11,432	0.737	0.607	0.005	4.000
*ΔNWC*	11,432	0.073	0.344	-4.388	7.047
*PPE*	11,432	0.271	0.173	0.003	0.774
*Board*	11,432	2.142	0.193	0.693	2.890
*lnAir*	11,432	10.107	7.314	0.000	18.618
*lnRail*	11,432	8.079	0.511	5.707	9.473
*lnRoad*	11,432	11.907	0.601	9.35	12.728
*lnWater*	11,432	7.871	2.704	0.000	10.102

### 4.2. Benchmark regression results

This paper uses the multi-period DID model to test Hypothesis 1, and the results are shown in Table 3. Three indexes of KZ, WW, and SA are selected as proxy variables of financing constraints, and the regression results are significant at the 5% level and the coefficient is negative, indicating that the opening of high-speed rail has a significant mitigation effect on corporate financing constraints. The regression coefficients of KZ index, WW index and SA index are -0.040, -0.004 and -0.003 respectively, indicating that the opening of high-speed rail can ease the financing constraints of enterprises at the level of 4.00%, 0.40% and 0.30%. At the same time, controlling for other modes of transportation such as flights, roads, railways and inland shipping, the results are not significant or positive, excluding the impact of other modes of transportation on corporate financing. Further verify the alleviating effect of the opening of high-speed railway on financing constraints of enterprises. This result supports Hypothesis 1 proposed in this paper.

**Table 3:** Benchmark regression results.

VARIABLES	(1)	(2)	(3)
	KZ	WW	SA
*HSR*	-0.040**	-0.004**	-0.003**
	(-2.17)	(-2.08)	(-2.03)
*ROA*	0.032	0.281***	0.046***
	(0.18)	(5.32)	(3.01)
*LEV*	0.946**	0.007	0.025**
	(2.51)	(0.69)	(2.08)
*Dual*	-0.041**	-0.003	-0.005*
	(-2.04)	(-1.07)	(-1.83)
*Balance*	-0.049**	-0.007**	-0.004**
	(-2.00)	(-2.31)	(-2.12)
*NWC*	-0.110***	-0.026***	-0.007***
	(-4.96)	(-7.14)	(-2.63)
*PPE*	0.061	0.017**	0.018**
	(0.79)	(2.15)	(2.07)
*Board*	-0.069	-0.008	-0.021**
	(-1.00)	(-1.11)	(-2.13)
*lnAir*	-0.004	-0.001**	-0.001**
	(-0.87)	(-2.18)	(-1.65)
*lnRail*	0.140**	0.023**	0.005
	(1.59)	(2.27)	(0.27)
*lnRoad*	-0.058	-0.001	-0.011*
	(-0.62)	(-0.10)	(-1.68)
*lnWater*	0.026*	0.000	0.002*
	(1.65)	(0.15)	(1.94)***
*Constant*	-0.459	-0.156**	4.029***
	(-1.32)	(-2.15)	(7.72)
*Firm*	YES	YES	YES
*Year*	YES	YES	YES
*N*	11,432	11,432	11,432
*R* ^*2*^	0.265	0.184	0.178

Notes

*** significance level at 1%

** significance level at 5%

* significance level at 10%. T-statistics is calculated based on standard errors clustered at the firm level. Same below.

### 4.3. Regression results of different property rights

In this paper, the whole sample is grouped according to whether it is the state-owned holding or not, and the results are shown in Table 4. From the regression results, it can be seen that in state-owned enterprises, the opening of high-speed railway has no significant effect on enterprise financing constraints, indicating that the opening of high-speed railway has a limited effect on state-owned holding enterprises’ financing constraints. However, in the grouped regression results of non-state-owned holding enterprises, the effect coefficients of the opening of high-speed railway on the financing constraints of enterprises are -7.00%, -0.80% and -0.50%, respectively. The regression coefficients of the three indexes are all negative and significant at the level of 5%. The results show that compared with state-owned holding enterprises, the opening of the high-speed railway has a more significant effect on alleviating financing constraints for non-state-owned holding enterprises. This result verifies hypothesis 2 proposed in this paper.

**Table 4:** Regression results of different property rights.

VARIABLES	KZ		WW		SA	
	(1)	(2)	(3)	(4)	(5)	(6)
	State-owned enterprise	Non-state-owned enterprise	State-owned enterprise	Non-state-owned enterprise	State-owned enterprise	Non-state-owned enterprise
*HSR*	-0.014	-0.070**	-0.003	-0.008**	-0.004	-0.005**
	(-0.41)	(-2.30)	(-0.72)	(-2.13)	(-0.86)	(-1.96)
P-value of Diff. in Coef	0.017**		0.005***		0.014**	
*ROA*	-0.364*	0.191	-0.270***	-0.267***	0.036	0.049***
	(-1.92)	(0.89)	(-9.44)	(-14.40)	(1.37)	(2.81)
*LEV*	0.942***	0.945***	0.019	0.016	0.051*	0.051***
	(7.22)	(8.89)	(1.13)	(1.25)	(1.83)	(3.55)
*Dual*	-0.056*	-0.036	0.001	-0.004	-0.010*	-0.004
	(-1.75)	(-1.51)	(0.16)	(-1.38)	(-1.73)	(-1.21)
*Balance*	-0.110***	-0.015	-0.014***	-0.005	-0.003	-0.003
	(-2.88)	(-0.52)	(-2.81)	(-1.45)	(-0.46)	(-0.73)
*ΔNWC*	-0.001	-0.147***	-0.036***	-0.024***	-0.003	-0.002
	(-0.05)	(-6.13)	(-3.95)	(-5.93)	(-0.33)	(-0.73)
*PPE*	0.080	0.108	0.058***	0.000	0.024	-0.005
	(0.69)	(1.28)	(3.59)	(0.03)	(0.88)	(-0.28)
*Board*	-0.040	-0.057	-0.001	-0.009	-0.001	-0.021*
	(-0.75)	(-0.72)	(-0.01)	(-0.90)	(-0.03)	(-1.76)
*lnAir*	-0.004	-0.003	-0.001*	-0.001**	-0.001	-0.001**
	(-0.86)	(-1.01)	(-1.89)	(-1.99)	(-1.32)	(-2.19)
*lnRail*	0.118	0.187*	0.020	0.029**	0.023**	0.023**
	(1.11)	(1.77)	(1.22)	(2.42)	(2.16)	(2.10)
*lnRoad*	0.124	0.114*	0.059***	0.011	0.032*	0.007
	(0.93)	(1.76)	(2.67)	(0.98)	(1.83)	(0.49)
*lnWater*	-0.033*	-0.024**	-0.014***	-0.003	-0.011	-0.002**
	(-1.66)	(-1.96)	(-2.80)	(-1.18)	(-1.09)	(-1.65)
*Constant*	-2.153*	-0.420	-0.781***	-0.068	3.488***	3.936***
	(-1.95)	(-1.10)	(-4.19)	(-0.89)	(11.38)	(13.57)
*Firm*	YES	YES	YES	YES	YES	YES
*Year*	YES	YES	YES	YES	YES	YES
*N*	3,356	8,076	3,356	8,076	3,356	8,076
*R* ^*2*^	0.210	0.337	0.191	0.165	0.181	0.186

### 4.4. Regression results for different industries

To examine the differences in the impact of the opening of high-speed rail on corporate financing constraints in different industries, this paper draws on the research of Lu Tong and Dang Yin [33], and according to the 2012 edition of the "Guidelines for the Classification of Listed Companies Industry", the industries in which the enterprises are located are classified according to the density of factors. It is divided into three categories: labor-intensive industries, capital-intensive industries and technology-intensive industries. The KZ index, WW index, and SA index are used as proxy variables for group regression. The results are shown in Table 5. In the labor-intensive industry grouping, the coefficients of the explanatory variable HSR are not significant, indicating that the opening of the high-speed rail does not have a significant effect on alleviating the financing constraints of labor-intensive industries. In the capital-intensive industry grouping, the coefficient score of the explanatory variable HSR is insignificant or significant at the 10% level, indicating that the opening of the high-speed rail has a limited impact on the financing constraints of capital-intensive enterprises. In the technology-intensive industry grouping, the coefficient of the explanatory variable HSR is significantly negative at the level of 5%, indicating that the opening of high-speed rail can have a greater impact on the degree of financing constraints of technology-intensive enterprises. This result verifies hypothesis 3 proposed in this paper.

**Table 5:** Regression results for different industries.

VARIABLES	*KZ*			*WW*			*SA*		
	(1)	(2)	(3)	(4)	(5)	(6)	(7)	(8)	(9)
	Labor intensive	Capital intensive	Technology intensive	Labor intensive	Capital intensive	Technology intensive	Labor intensive	Capital intensive	Technology intensive
*HSR*	-0.027	-0.017	-0.042**	-0.004	-0.003	-0.002**	-0.006	-0.009*	-0.003**
	(-0.53)	(-0.46)	(-2.17)	(-0.63)	(-0.55)	(-2.53)	(-1.55)	(-1.69)	(-2.52)
P-value of Diff. in Coef	0.014**			0.008***			0.014**		
*ROA*	0.307*	0.399**	0.134	0.243***	0.309***	0.249***	0.050**	0.042**	0.068***
	(1.01)	(2.37)	(0.74)	(8.42)	(7.17)	(7.17)	(2.24)	(2.18)	(3.62)
*LEV*	0.932***	0.810***	1.174***	0.013	0.024	0.030*	0.029	0.009	0.056***
	(3.74)	(5.22)	(8.91)	(0.63)	(1.53)	(1.84)	(1.13)	(0.45)	(2.79)
*Dual*	-0.027	-0.096***	-0.015	-0.002	-0.005	-0.001	-0.001	-0.008*	-0.001
	(-0.68)	(-2.98)	(-0.53)	(-0.45)	(-0.98)	(-0.25)	(0.11)	(-1.66)	(-0.29)
*Balance*	-0.024	-0.106**	-0.046*	-0.005	-0.008	-0.007*	-0.006**	-0.008**	-0.002
	(-0.55)	(-2.47)	(-1.69)	(-0.74)	(-1.40)	(-1.75)	(-1.97)	(-2.20)	(-0.33)
*ΔNWC*	-0.110*	-0.100**	-0.132***	-0.023***	-0.021***	-0.031***	-0.002	-0.010	-0.002
	(-1.87)	(-2.45)	(-4.11)	(-3.68)	(-3.50)	(-5.49)	(-0.60)	(-1.32)	(-0.70)
*PPE*	0.000	0.064	0.247***	0.056**	0.018	0.018	0.020	0.047**	0.021*
	(0.00)	(0.43)	(2.59)	(2.57)	(1.07)	(1.11)	(0.79)	(2.18)	(1.95)
*Board*	-0.005	-0.027	-0.111	-0.023*	-0.007	-0.021*	-0.005	-0.019	-0.028*
	(-0.04)	(-0.49)	(-1.02)	(-1.94)	(-0.71)	(-1.80)	(-0.27)	(-1.13)	(-1.75)
*lnAir*	-0.011*	-0.004	-0.007	-0.001	-0.002**	-0.001	-0.001**	-0.001	-0.001
	(-1.85)	(-0.71)	(-1.40)	(-0.72)	(-2.29)	(-1.01)	(-1.99)	(-0.50)	(-0.71)
*lnRail*	0.047	0.038	0.093	0.021	0.016	0.022**	0.018	0.059***	0.013
	(0.32)	(0.19)	(0.81)	(1.21)	(0.83)	(2.33)	(0.84)	(2.94)	(0.62)
*lnRoad*	-0.049	-0.085	-0.027	-0.007	-0.001	-0.017	-0.042**	-0.048**	-0.003
	(-0.30)	(-0.47)	(-0.18)	(-0.39)	(-0.07)	(-1.04)	(-2.11)	(-2.49)	(-0.07)
*lnWater*	0.009	0.012	0.077**	0.002	0.001	0.001	0.004*	0.004*	0.004*
	(0.25)	(0.43)	(2.35)	(0.43)	(0.35)	(0.27)	(1.93)	(1.75)	(1.69)
*Constant*	0.116	-0.457	-0.868	-0.109	-0.079	-0.328**	4.232***	3.921***	3.973***
	(0.17)	(-0.39)	(-0.68)	(-0.90)	(-0.77)	(-2.14)	(9.50)	(10.10)	(14.69)
*Firm*	YES	YES	YES	YES	YES	YES	YES	YES	YES
*Year*	YES	YES	YES	YES	YES	YES	YES	YES	YES
*N*	2,850	3,740	4,842	2,850	3,740	4,842	2,850	3,740	4,842
*R* ^*2*^	0.232	0.241	0.350	0.127	0.175	0.252	0.182	0.161	0.101

### 4.5. Regression results in different regions

Coastal areas are defined as areas with coastlines in China Statistical Yearbook of Oceanography [51]. According to this definition, China now has 9 coastal cities, 1 autonomous region and 2 municipalities. In this paper, the samples are divided into coastal areas and inland areas according to the city where the enterprise office is located, and the results are shown in Table 6. In the grouping of coastal cities, KZ index, WW index and SA index are used as proxy variables respectively, and the results of explanatory variables HSR are not significant, indicating that the opening of high-speed railway has limited alleviating effect on financing constraints of enterprises in coastal cities. In the grouped regression results of the inland region, the explanatory variable HSR is significantly negative at the level of 5%, respectively, indicating that the opening of the high-speed railway can effectively alleviate the financing constraints of inland enterprises. This result verifies hypothesis 4 proposed in this paper.

**Table 6:** Regression results in different regions.

VARIABLES	KZ		WW		SA	
	(1)	(2)	(3)	(4)	(5)	(6)
	Coastal area	Inland area	Coastal area	Inland area	Coastal area	Inland area
*HSR*	-0.032	-0.034**	-0.007	-0.002**	0.001	-0.003**
	(-1.04)	(-2.18)	(-1.03)	(-2.41)	(0.38)	(-2.44)
P-value of Diff. in Coef	0.043**		0.006***		0.005***	
*ROA*	-0.004	-0.022	-0.276***	-0.296***	-0.061***	0.023
	(-0.03)	(-0.07)	(-13.69)	(-11.43)	(-3.38)	(0.88)
*LEV*	0.875***	0.954***	0.004	0.006	0.058***	-0.021
	(7.09)	(6.22)	(0.26)	(0.36)	(3.81)	(-0.85)
*Dual*	-0.015	-0.082**	-0.004	-0.001	-0.006	-0.003
	(-0.67)	(-2.36)	(-1.11)	(-0.20)	(-1.55)	(-0.57)
*Balance*	-0.021	-0.098**	-0.004	-0.014***	-0.002	-0.012*
	(-0.64)	(-2.53)	(-0.87)	(-3.20)	(-0.48)	(-1.89)
*ΔNWC*	-0.152***	-0.063	-0.029***	-0.021***	-0.005**	-0.009
	(-5.55)	(-1.51)	(-5.24)	(-5.56)	(-1.97)	(-1.35)
*PPE*	0.146*	0.032	0.013	0.029*	0.003	0.035**
	(1.75)	(0.26)	(1.43)	(1.67)	(0.14)	(2.21)
*Board*	0.002	-0.135*	-0.004	-0.007	0.021**	0.017**
	(0.02)	(-1.68)	(-0.56)	(-0.75)	(2.52)	(1.99)
*lnAir*	-0.001	-0.006	-0.001	-0.002***	-0.000	-0.002*
	(-0.37)	(-1.13)	(-1.00)	(-2.84)	(-0.54)	(-1.91)
*lnRail*	0.164*	0.058	0.019	0.020*	0.002	0.026**
	(1.74)	(0.35)	(1.27)	(1.90)	(0.05)	(2.53)
*lnRoad*	-0.118	-0.133*	-0.008	-0.001	-0.025**	-0.013
	(-0.83)	(-1.91)	(-0.57)	(-0.11)	(-2.20)	(-0.51)
*lnWater*	0.016	0.013	0.003*	0.003*	0.004**	0.007**
	(0.38)	(0.64)	(1.91)	(1.67)	(1.90)	(2.38)
*Constant*	-0.065	-0.808*	-0.187***	-0.130**	4.087***	3.964***
	(-0.06)	(-1.86)	(-2.64)	(-2.57)	(4.29)	(6.45)
*Firm*	YES	YES	YES	YES	YES	YES
*Year*	YES	YES	YES	YES	YES	YES
*N*	7,261	4,171	7,261	4,171	7,261	4,171
*R* ^*2*^	0.305	0.232	0.158	0.235	0.194	0.160

### 4.6. Robustness test

#### 4.6.1. Parallel trend test

The important prerequisite for using the difference-in-differences is that the treatment group and the control group must have a common trend before the policy is implemented, that is, to meet the parallel trend test. In this paper, the regression method is used to test the parallel trend. In this paper, the regression method is used to test the parallel trend, and the year when the high-speed rail is opened at the company’s location is determined as the base year, D_1 is the year before the high-speed rail was opened, D_2+ is the year more than one year before the high-speed rail was opened, D0 is the year when the high-speed rail was opened, D1 is the year after the opening of the high-speed railway, D2 is the year after the opening of the high-speed railway for two years, and D5 is the year after the opening of the high-speed railway for five years. These six variables are added to the model (1) for fixed effect regression. If the sample data meets the parallel trend assumption, the results of D_1 and D_2+ should not be significant, and D0, D1, D2 and D5will be significantly negative, indicating that the difference between the experimental group and the control group is caused by the exogenous event of the high-speed rail opening.

The specific regression results are shown in Table 7. Using KZ, WW, and SA indexes as proxy variables, the results of D_1 and D_2+ regression coefficients in the year before the opening of the high-speed rail were not significant. Taking the WW index as an example, the coefficient of D0 is significantly negative at the level of 1% in the year when the high-speed rail was opened, and the coefficients of D1, D2 and D5 are also significantly negative at the level of 5%. The results show that there is no trait difference between the experimental group and the control group before the opening of the high-speed rail, and the difference between the two after the opening of the high-speed rail is caused by the exogenous event of the opening of the high-speed rail, and the impact is not temporary. This result passes the parallel trend test.

**Table 7:** Results of the parallel trend test.

VARIABLES	(1)	(2)	(3)
	KZ	WW	SA
*D_1*	-0.036	-0.001	-0.002
	(-1.10)	(-0.20)	(-0.60)
*D_2+*	-0.025	-0.004	-0.004
	(-0.87)	(-0.67)	(-1.19)
*D0*	-0.014*	-0.004***	-0.001**
	(-1.77)	(-2.64)	(-2.32)
*D1*	-0.001**	-0.002**	-0.005**
	(-1.97)	(-2.13)	(-2.18)
*D2*	-0.020**	-0.001**	-0.002**
	(-1.96)	(-2.04)	(-2.51)
*D5*	-0.063***	-0.007**	-0.009**
	(-2.70)	(-2.27)	(-1.99)
*ROA*	-0.660***	-0.308***	0.110***
	(-2.66)	(-7.86)	(3.83)
*LEV*	0.917***	-0.060***	0.020
	(4.94)	(-2.16)	(0.77)
*Dual*	0.001	0.000	-0.009*
	(0.04)	(0.08)	(-1.91)
*Balance*	-0.068*	-0.009*	-0.011*
	(-1.66)	(-1.94)	(-1.82)
*ΔNWC*	0.088*	-0.049***	0.009*
	(1.74)	(-4.30)	(1.93)
*PPE*	0.055	0.038**	0.014
	(0.44)	(1.96)	(0.65)
*Board*	-0.035	-0.015	-0.019**
	(-0.34)	(-1.20)	(-2.31)
*lnAir*	-0.009**	-0.001	-0.001**
	(-2.46)	(-1.07)	(-2.17)
*lnRail*	0.134	0.024	0.004
	(0.095)	(1.30)	(0.16)
*lnRoad*	-0.102	-0.001	-0.021*
	(-0.42)	(-0.04)	(-1.67)
*lnWater*	0.047**	0.003	0.007*
	(2.13)	(0.43)	(1.84)
*Constant*	-2.332**	-0.121	3.953***
	(-2.07)	(-0.43)	(12.23)
*Firm*	YES	YES	YES
*Year*	YES	YES	YES
*N*	3,173	3,173	3,173
*R* ^*2*^	0.193	0.189	0.106

#### 4.6.2. Placebo test

To eliminate the influence of potential missing variables, the paper conducted a placebo test, lagging the opening year of the high-speed rail by 2–4 years, and redefining the explanatory variable HSR. If the "pseudo-high-speed rail opening" has a significant impact on corporate financing constraints, it means that the original conclusion may be caused by some missing variables. On the contrary, it shows that the opening of the high-speed rail does have an impact on corporate financing constraints. In this paper, the regression of high-speed railway opening time after 2–4 years lag is carried out, and the results are shown in Table 8. In the table, L2, L3 and L4 represent 2–4 years lag respectively. The results show that the coefficients of HSR are not significant, which explains the effect of high-speed railway opening on financing constraints of enterprises from the side.

**Table 8:** Placebo test results.

VARIABLES	L2			L3			L4		
	(1)	(2)	(3)	(4)	(5)	(6)	(7)	(8)	(9)
	KZ	WW	SA	KZ	WW	SA	KZ	WW	SA
*HSR*	0.017	0.005	-0.005	0.011	0.001	-0.006	0.042	0.004	-0.009
	(0.69)	(1.61)	(-1.30)	(0.47)	(0.28)	(-0.88)	(1.51)	(1.00)	(-0.99)
*ROA*	-0.028	-0.275***	0.022	-0.109	-0.261***	-0.014	-0.098	-0.244***	0.007
	(-0.15)	(-15.96)	(1.49)	(-0.68)	(-14.71)	(-0.95)	(-0.55)	(-13.61)	(-0.47)
*LEV*	0.966***	-0.001**	0.009	1.000***	0.002**	-0.005	0.995***	0.001	-0.005
	(3.51)	(-2.05)	(1.53)	(3.44)	(2.21)	(-1.42)	(2.94)	(1.11)	(1.35)
*Dual*	-0.047**	-0.004	-0.002	-0.059***	-0.002	-0.002	-0.076***	-0.001	-0.002
	(-2.19)	(-1.18)	(-0.55)	(-2.83)	(-0.63)	(-0.67)	(-3.36)	(-0.40)	(-0.47)
*Balance*	-0.042	-0.006*	0.007*	-0.048**	-0.007**	0.006	-0.044	-0.009**	0.007
	(-1.57)	(-1.85)	(1.74)	(-2.07)	(-1.98)	(1.59)	(-1.63)	(-2.10)	(1.49)
*ΔNWC*	-0.053	-0.031***	0.020***	-0.031	-0.033***	0.015***	-0.083**	-0.026***	0.014***
	(-1.59)	(-5.98)	(4.34)	(-0.81)	(-5.87)	(3.13)	(-1.96)	(-5.15)	(2.95)
*PPE*	-0.035	0.016	0.008	-0.135	0.014	0.005	-0.118	0.011	0.008
	(-0.43)	(1.36)	(0.39)	(-1.63)	(1.05)	(0.19)	(-1.23)	(0.74)	(0.36)
*Board*	-0.048	-0.010	-0.018*	-0.012	-0.007	0.020*	-0.034	-0.017*	-0.016
	(-0.68)	(-1.16)	(-1.69)	(-0.16)	(-0.76)	(1.87)	(-0.43)	(-1.68)	(-1.55)
*lnAir*	-0.005	-0.001***	-0.001	-0.005	-0.001**	-0.000	-0.000	-0.001	-0.000
	(-1.21)	(-2.90)	(-0.94)	(-1.14)	(-2.26)	(-0.73)	(-0.09)	(-1.60)	(-0.20)
*lnRail*	0.101	0.021*	0.019	0.072	0.023*	0.026	0.010	0.016	0.026
	(0.88)	(1.79)	(1.11)	(0.65)	(1.75)	(1.58)	(0.09)	(1.08)	(1.62)
*lnRoad*	0.004	0.007*	0.007	0.017**	0.005	0.014*	0.087	0.004	0.013*
	(0.04)	(1.67)	(0.41)	(2.15)	(0.38)	(1.78)	(0.90)	(0.31)	(1.74)
*lnWater*	0.022*	0.002	0.004	0.017*	0.002	0.005	0.013	0.000	0.005**
	(1.87)	(0.90)	(1.04)	(1.70)	(0.65)	(1.19)	(0.69)	(0.10)	(2.05)
*Constant*	-0.917	-0.197***	3.972***	-0.856	-0.199***	3.971***	-1.183**	-0.123	3.991***
	(-1.18)	(-2.64)	(3.02)	(-1.21)	(-2.62)	(3.35)	(-2.08)	(-1.63)	(3.40)
*Firm*	YES	YES	YES	YES	YES	YES	YES	YES	YES
*Year*	YES	YES	YES	YES	YES	YES	YES	YES	YES
*N*	8,547	8,547	8,547	7,373	7,373	7,373	6,340	6,340	6,340
*R* ^*2*^	0.226	0.172	0.164	0.230	0.156	0.151	0.240	0.121	0.135

#### 4.6.3. Replace the explained variable

(1) Cash—cash flow sensitivity model. In the robustness test, the proxy variables of financing constraints are replaced with the improved cash-cash flow sensitivity model proposed by Almeida et al. [20]. When a company has financing constraints, it will reserve a set of cash. The greater the degree of financing constraints the company faces, the stronger the cash-cash flow sensitivity. Establish the benchmark model (5) as:

$$\Delta Cash_{i,t}=\alpha_{0}+\alpha_{1}CF_{i,t}+\alpha_{2}Size_{i,t}+\alpha_{3}\Delta STD_{i,t}+\alpha_{4}\Delta NWC_{i,t}+\alpha_{5}Growth_{i,t}+\alpha_{6}Expend_{i,t}+\varepsilon_{i,t}$$ (5)

In model (5), the coefficient *α*_1_ is used to judge whether the company has financing constraints. At the same time, to verify Hypothesis 1, this paper adds the interaction item of corporate cash flow and the opening of high-speed rail based on the benchmark model, and further constructs an extended model. Model (6) is as follows:

$$\Delta Cash_{i,t}=\alpha_{0}+\alpha_{1}CF_{i,t}+\alpha_{2}Size_{i,t}+\alpha_{3}\Delta STD_{i,t}+\alpha_{4}\Delta NWC_{i,t}+\alpha_{5}Growth_{i,t}+\alpha_{6}Expend_{i,t}+\alpha_{7}CF_{i,t}*HSR_{i,t}+\varepsilon_{i,t}$$ (6)

If Hypothesis 1 holds, that is, the opening of high-speed rail can alleviate corporate financing constraints, then the interaction coefficient *α*_7_ in model (6) should be significantly negative. The main variables are described in Table 9. The regression results of the model are shown in Table 10.

**Table 9:** Description of the main variables.

Variable symbol	Variable definitions
*ΔCash*	Increase in cash and cash equivalents/Total assets at the beginning of period
*CF*	Net cash flow from operating activities/Total assets at the beginning
*Expend*	Long term capital expenditure/total assets at the beginning
*Size*	The natural logarithm of total assets at the end of the period
*ΔSTD*	Increase in current liabilities/total assets at the beginning of the period
*ΔNWC*	Increase in net working capital/total assets at the beginning of the period
*Growth*	Main business income growth rate
*LEV*	Total liabilities/total assets at the end of the period
*Board*	The natural logarithm of the number of board members
*Age*	The natural logarithm of the age of the company

**Table 10:** Regression results of cash-cash flow sensitivity model.

Variable	Model (1)	Model (2)
*CF*	0.339***	0.351***
	(9.33)	(7.49)
*Size*	0.062***	0.062***
	(15.56)	(15.58)
*ΔSTD*	0.014***	0.014***
	(3.03)	(3.04)
*ΔNWC*	0.053*	0.054*
	(1.91)	(1.93)
*Growth*	0.014***	0.014***
	(2.60)	(2.60)
*Expend*	0.091*	0.093*
	(1.95)	(1.94)
HSR*CF		-0.025**
		(-2.42)
*Constant*	-1.291***	-1.291***
	(-15.86)	(-15.86)
*Firm*	YES	YES
*Year*	YES	YES
*N*	11,432	11,432
*R* ^*2*^	0.173	0.173

The coefficient *α*_*i*_ of CF in the benchmark model (5) is significantly positive at the 1% level, indicating that companies generally have financing constraints. At the same time, the coefficient of interaction item in the extended model (6) is -0.025, and it is significantly negative at the 5% level. It shows that the opening of the high-speed railway can alleviate enterprise financing constraints to a 2.5% degree. Hypothesis 1 was further tested.

(2) Investment-cash flow sensitivity model. Fazzari et al. proposed to use the investment-cash flow sensitivity coefficient to measure the degree of corporate financing constraints [42]. Based on the investment perspective, this method constructs the investment-cash flow sensitivity coefficient, which reflects the satisfaction degree of the cash flow to the investment. At the same time, it also considers information asymmetry and agency problems. Many subsequent scholars also use it as a measure of corporate financing constraints. Drawing on the research of Custódio and Metzger, Jiang Fuxiu et al. [52, 53], this paper constructs the following empirical model (7):

$$Expend_{i,t}=\alpha_{0}+\alpha_{1}CF_{i,t}+\alpha_{2}HSR_{i,t}*CF_{i,t}+\alpha_{3}HSR_{i,t}+Controls+\varepsilon_{i,t}$$ (7)

The explained variable *Expend*_*i*,*t*_ is the investment variable, which is measured by capital expenditure. *CF*_*i*,*t*_ is the cash flow of the enterprise, and *HSR*_*i*,*t*_ is the core explanatory variable of this paper, which measures whether the office location of enterprise i has opened a high-speed rail in year t. Selects the enterprise size (*Size*), sales growth rate (*Growth*), asset-liability ratio (*LEV*), board size (*Board*), and firm age (*Age*) as control variables. The specific variable descriptions are shown in Table 9. If Hypothesis 1 is established, that is, the opening of high-speed rail can alleviate the financing constraints of enterprises, then the coefficient *α*_2_ of the interaction term in model (7) should be significantly negative. The specific regression results are shown in Table 11.

**Table 11:** Regression results of investment-cash flow sensitivity model.

VARIABLES	(1)
	Expend
*CF*	0.036**
	(2.34)
HSR*CF	-0.068***
	(-3.34)
*HSR*	-0.008**
	(-2.52)
*Size*	0.007***
	(6.69)
*Growth*	0.010***
	(6.28)
*LEV*	-0.016**
	(-2.21)
*Balance*	0.006**
	(2.18)
*Age*	-0.067***
	(-7.04)
*Constant*	0.097**
	(2.58)
*Firm*	YES
*Year*	YES
*N*	11,432
*R* ^*2*^	0.158

The regression results of model (7) show that the coefficient *α*_1_ of CF is significantly positive at the 1% level, which has a significant positive impact on corporate investment. The coefficient of high-speed rail opening (HSR) is significantly negative at the level of 1%, while the coefficient of the interaction term (HSR*CF) is significantly negative at the level of 1%, which indicates that the opening of high-speed rail can reduce the level of investment of enterprises, which further verifies Hypothesis 1 proposed in this paper.

## 5. Influence mechanism analysis

Based on the previous analysis, the opening of high-speed rail can reduce the degree of information asymmetry between enterprises and their stakeholders, alleviate agency conflicts of enterprises, thereby alleviating the financing constraints of enterprises. Based on the level of information asymmetry and the level of agency conflicts, this paper conducts a group regression of the samples and analyzes the path through which the opening of high-speed rail affects the degree of financing constraints of enterprises.

### 5.1. Mechanism inspection based on information asymmetry

The opening of high-speed rail can reduce the degree of information asymmetry between enterprises and investors, thereby easing the financing constraints of enterprises. Drawing on the practices of Wu Weixing et al. and O’Neill et al. [44, 45], this paper selects the shareholding ratio of institutional investors (*Institution*) as the index to measure the degree of information asymmetry. To obtain better returns and safeguard their own interests, institutional investors will actively participate in corporate governance, supervise the behavior of corporate executives, and improve corporate governance, thereby reducing management’s opportunistic behavior and increasing information transparency and disclosure quality [54, 55]. The higher the shareholding ratio of institutional investors, the stronger the role of supervision on listed companies, and the degree of information asymmetry of enterprises will be reduced. Taking the median of the shareholding ratio of institutional investors as the cut-off point, the samples are divided into two groups: "high degree of information asymmetry" and "low degree of information asymmetry". The KZ index, WW index, and SA index are used as the proxy variables of financing constraints for group regression to analyze the influence path of the opening of high-speed rail on corporate financing constraints. The results are shown in Table 12. According to the results in the table, it can be found that the coefficient of the explanatory variable HSR is more significant in the group with a high degree of information asymmetry. Taking the regression result of the SA index as an example, in the group with a high degree of information asymmetry, the coefficient of HSR is significantly negative at the 1% level. However, the coefficient of HSR is not significant in the group with low information asymmetry. This indicates that the opening of high-speed railway has a more significant easing effect on the financing constraints of enterprises with high degree of information asymmetry, which further verifies that the opening of the high-speed railway can reduce the degree of information asymmetry of enterprises and thus alleviate the financing constraints of enterprises.

**Table 12:** Test results based on information asymmetry mechanism.

VARIABLES	KZ		WW		SA	
	(1)	(2)	(3)	(4)	(5)	(6)
	Low information asymmetry group	High information asymmetry group	Low information asymmetry group	High information asymmetry group	Low information asymmetry group	High information asymmetry group
*HSR*	-0.033	-0.070**	-0.000	-0.007**	-0.002	-0.009***
	(-0.93)	(-2.26)	(-0.19)	(-2.13)	(-0.45)	(-2.79)
P-value of Diff. in Coef	0.046**		0.006***		0.033**	
*ROA*	-0.041	-0.344**	-0.227***	-0.294***	-0.022	-0.077***
	(-0.20)	(-2.37)	(-11.76)	(-11.54)	(-1.20)	(-3.52)
*LEV*	0.884***	0.994***	0.034**	0.026*	0.052***	0.019
	(6.42)	(6.90)	(2.39)	(1.91)	(3.01)	(1.00)
*Dual*	-0.029	-0.035	-0.000	-0.009**	-0.001	-0.010**
	(-1.01)	(-1.22)	(-0.17)	(-2.02)	(-0.18)	(-2.12)
*Balance*	-0.070**	-0.045*	-0.005	-0.012**	0.004	-0.001
	(-2.41)	(-1.74)	(-1.12)	(-2.44)	(1.00)	(-0.19)
*ΔNWC*	-0.090***	-0.080**	-0.015***	-0.033***	-0.009***	-0.004
	(-2.72)	(-2.32)	(-2.91)	(-6.37)	(-3.71)	(-1.02)
*PPE*	0.144**	0.256***	0.010	0.048***	0.039**	0.017*
	(2.00)	(2.63)	(0.65)	(3.64)	(2.29)	(1.85)
*Board*	-0.002	-0.067	-0.007	0.010	-0.033***	-0.008
	(-0.03)	(-0.87)	(-0.82)	(0.87)	(-2.96)	(-0.42)
*lnAir*	-0.002	-0.009**	-0.000	-0.001*	-0.001	-0.000
	(-0.38)	(-2.23)	(-0.07)	(-1.65)	(-0.55)	(-1.21)
*lnRail*	0.041	0.239**	0.027*	0.006	0.011	0.028**
	(0.28)	(2.20)	(1.77)	(0.41)	(1.13)	(2.39)
*lnRoad*	-0.134	-0.195***	-0.008	-0.023*	-0.012*	-0.038***
	(-0.96)	(-2.62)	(-0.53)	(-1.67)	(-1.67)	(-2.91)
*lnWater*	0.006	0.023*	0.002	0.002	0.004**	0.001
	(0.96)	(1.83)	(0.77)	(0.46)	(2.13)	(1.28)
*Constant*	-1.056*	0.289	-0.098*	-0.312***	3.776***	4.103***
	(-1.77)	(0.42)	(-1.85)	(-3.26)	(17.43)	(12.22)
*Firm*	YES	YES	YES	YES	YES	YES
*Year*	YES	YES	YES	YES	YES	YES
*N*	5,698	5,734	5,698	5,734	5,698	5,734
*R* ^*2*^	0.122	0.138	0.135	0.169	0.112	0.158

In order to further test the impact of field research by institutional investors on the relationship between the opening of high-speed railway and financing constraints of enterprises, and whether there are differences in the impact of different types of institutional investors. This paper collects and sort out the relevant data of whether institutional investors conducted field research and the shareholding ratio of different types of institutional investors. This paper examines the influence of the field survey of institutional investors and the shareholding of different types of institutional investors on the relationship between the opening of high-speed railway and the financing constraints of enterprises, thus providing more favorable support for the hypothesis that the opening of high-speed railway affects the possibility of site visits, and thus influences the financing constraints. For institutional investors, in enterprises with a higher proportion of shares held by institutional investors, the stock price fluctuations and other performance of enterprises are more closely related to the direct interests of institutional investors. In order to safeguard their own interests, institutional investors will be more motivated to conduct field research on enterprises to obtain more information and enhance the governance effect on enterprises. The results also show that, in the group conducted field research by institutional investors, there is a significant negative correlation between the opening of high-speed railway and the financing constraints of enterprises, confirming that the opening of high-speed railway can reduce the degree of information asymmetry through the field research by institutional investors, and thus alleviating the financing constraints of enterprises. At present, institutional investors are divided into 10 types: Fund, QFII, Broker, Insurance, Security Fund, Entrust, Finance, Bank, Non-Finance and Other institutions. Among enterprises held by different types of institutional investors, Broker investors, Entrust investors, Finance investors, Bank investors and other institutional investors will be affected by the opening of high-speed rail. This result may be due to the different frequency of field research and preference of travel tools for different types of institutional investors. Broker investors, Entrust investors, Finance investors and Bank investors mostly manage funds from the standpoint of agents. They hope to obtain more "soft information" through field research and enhance the understanding of target enterprises to make more accurate investment decisions. The opening of high-speed rail reduces the time and travel cost, making it easier for institutional investors to visit enterprises and promoting their field research to a certain extent. Insurance and social security funds, on the other hand, pay more attention to the preservation and increment of funds, and have relatively low demand and frequency for field research. Different types of institutional investors have different positions and business preferences, leading to differences in their impact on the opening of high-speed railway and enterprise financing. The results are no longer shown in this article as supplement.

### 5.2. Mechanism inspection based on agency problem

The opening of high-speed rail can alleviate agency conflicts of enterprises and ease the financing constraints of enterprises. Based on the above analysis, listed companies usually have two kinds of agency problems, namely, the agency problem between shareholders and management and the agency problem between controlling shareholders and minority shareholders. In order to test whether the opening of high-speed railway will have an impact on the two types of agency problems of enterprises, this paper selects different indicators to measure the two types of agency conflicts of enterprises.

Drawing on the practices of Ang et al. and Dai Yiyi et al. [46, 47], the operating expense ratio (*Cost*) is selected as the index to measure the first type of agency conflicts of enterprises in this paper, which reflects the waste caused by excessive in-service consumption by managers. Moreover, the consumption beyond the budget and other agency costs spent by managers can be directly evaluated because they are controlled by agents [56]. The higher the operating expense ratio, the higher the first type of agency conflicts of the company. Taking the median of the operating expense ratio as the cut-off point, the samples are divided into two groups: "high agency conflicts " and "low agency conflicts ". In addition, KZ index, WW index and SA index are successively used as proxy variables of financing constraints for grouping regression, and the impact path of the opening of high-speed railway on financing constraints of enterprises is analyzed, as shown in Table 13. From the data in the table, it can be found that the coefficient of the explanatory variable HSR is more significant in the group with high agency conflicts. Take the SA index as a proxy variable as an example. In the group with high agency conflicts, the explanatory variable HSR is significantly negative at the 5% level, while in the group with low information asymmetry, the coefficient of HSR is not significant, indicating that the opening of high-speed rail has a more significant effect on alleviating financing constraints for companies with higher agency conflicts. It further verifies the impact path that the opening of high-speed rail can reduce the first type of agency problem of enterprises and thus ease the financing constraints of enterprises.

**Table 13:** Test results of the first type of agency problem mechanism.

VARIABLES	KZ		WW		SA	
	(1)	(2)	(3)	(4)	(5)	(6)
	Low agency conflicts group	High agency conflicts group	Low agency conflicts group	High agency conflicts group	Low agency conflicts group	High agency conflicts group
*HSR*	-0.003	-0.076***	-0.003	-0.006**	-0.001	-0.004**
	(-0.07)	(-2.83)	(-0.72)	(-2.14)	(-0.32)	(-2.10)
P-value of Diff. in Coef	0.024**		0.016**		0.009***	
*ROA*	0.182	0.298**	0.208***	0.349***	0.040**	0.052
	(0.81)	(2.31)	(11.46)	(12.60)	(2.24)	(1.95)
*LEV*	1.056***	1.022***	0.040***	0.030**	0.043**	0.008
	(7.91)	(9.18)	(2.62)	(2.16)	(2.57)	(0.51)
*Dual*	-0.030	-0.040*	-0.004	-0.001	-0.001	-0.008**
	(-1.17)	(-1.66)	(-1.24)	(-0.27)	(-0.28)	(-2.02)
*Balance*	-0.061*	-0.052*	-0.004	-0.009**	-0.008*	-0.009**
	(-1.90)	(-1.94)	(-0.95)	(-2.03)	(-1.89)	(-1.98)
*ΔNWC*	-0.127***	-0.063**	-0.026***	-0.022***	-0.006*	-0.009**
	(-4.04)	(-2.42)	(-6.88)	(-2.90)	(-1.85)	(-2.23)
*PPE*	0.091	0.139**	0.023*	0.019	0.021*	-0.013*
	(0.79)	(2.17)	(1.88)	(1.37)	(1.73)	(-1.64)
*Board*	-0.027	-0.039	-0.016*	-0.000	-0.039***	-0.008
	(-0.26)	(-0.70)	(-1.93)	(-0.06)	(-3.06)	(-0.92)
*lnAir*	-0.005	-0.002	-0.000	-0.001*	-0.000	-0.000
	(-0.85)	(-0.58)	(-0.58)	(-1.88)	(0.26)	(-0.74)
*lnRail*	0.009	0.165**	0.012	0.027**	0.016	0.038**
	(0.07)	(2.00)	(0.75)	(2.46)	(0.70)	(2.03)
*lnRoad*	-0.032	-0.000	-0.023**	-0.007	-0.024**	-0.029**
	(-0.19)	(-0.02)	(-1.94)	(-0.62)	(-1.92)	(-1.73)
*lnWater*	-0.038**	-0.011	-0.001	-0.004*	-0.001	-0.002
	(-2.28)	(-0.48)	(-0.33)	(-1.71)	(-0.23)	(-0.52)
*Constant*	0.203	-1.349**	-0.115*	-0.251***	4.213***	3.991***
	(0.14)	(-2.00)	(-1.85)	(-2.61)	(7.63)	(5.84)
*Firm*	YES	YES	YES	YES	YES	YES
*Year*	YES	YES	YES	YES	YES	YES
*N*	5,721	5,711	5,721	5,711	5,721	5,711
*R* ^*2*^	0.320	0.234	0.168	0.177	0.103	0.176

Referring to the research of Wei and Zhang [48], this paper selects the separation degree of two rights, namely the ownership and control right of the actual controller (Divergence), as the measurement index of the second type of agency problem. For listed companies, the most important ownership is equity ownership, which is the most distinct difference from ordinary companies. In China’s listed companies, share ownership tends to be relatively concentrated by a major shareholder, and the shares of the major shareholder are held by the major shareholder behind him/her, and so on many times. Finally, by means of pyramidal shareholding structure, some ultimate controlling shareholder can control the listed company with higher voting rights and less ownership. Drawing on Claessens et al. [57], this paper regards the cash flow right of the ultimate controlling shareholder as its ownership, and the voting right as its control right. According to the equity control chain, the difference between the two rights is calculated to measure the severity of the agency problem between shareholders. The severity of the second type of agency problem increases with the divergence between ultimate shareholder control and ownership. Therefore, this paper uses 0 as the cut-off point to group sample data. The sample from which Divergence greater than 0 (that is, there is a disagreement between control and ownership) is the high-agent conflicts group; The sample from Divergence equals 0 (that is, the control and ownership are consistent) is the low-agency conflicts group, with the results shown in Table 14. In the high agency conflicts group, HSR is significantly negative at the 5% level, indicating that there is a significant negative correlation between the opening of high-speed railway and financing constraints of enterprises; while in the low agency conflicts group, there is a negative correlation but the result is not significant, indicating that the opening of high-speed railway can alleviate the agency problem among shareholders to some extent. It is verified that the opening of high-speed railway can reduce the second type of agency problem of enterprises and thus alleviate the financing constraints of enterprises.

**Table 14:** The test results of the second type of agency problem mechanism.

VARIABLES	KZ		WW		SA	
	(1)	(2)	(3)	(4)	(5)	(6)
	Low agency conflicts group	High agency conflicts group	Low agency conflicts group	High agency conflicts group	Low agency conflicts group	High agency conflicts group
*HSR*	-0.022	-0.026***	-0.002	-0.012***	-0.011	-0.012**
	(-0.61)	(-2.70)	(-0.39)	(-2.83)	(-1.23)	(-2.18)
P-value of Diff. in Coef	0.008***		0.024**		0.001***	
*ROA*	0.130	0.387*	0.290***	0.293***	0.070***	0.040**
	(0.56)	(1.69)	(13.82)	(9.79)	(3.72)	(2.13)
*LEV*	0.934***	1.275***	0.005	0.030*	0.016	0.091***
	(7.56)	(9.70)	(0.37)	(1.94)	(0.94)	(5.18)
*Dual*	-0.042	-0.037	-0.001	-0.003	-0.007*	-0.005
	(-1.60)	(-1.17)	(-0.16)	(-0.83)	(-1.76)	(-1.29)
*Balance*	-0.009	-0.120***	-0.000	-0.014***	-0.000	-0.009
	(-0.27)	(-2.99)	(-0.09)	(-2.81)	(-0.08)	(-1.59)
*ΔNWC*	-0.112***	-0.166***	-0.029***	-0.005	-0.007**	-0.010***
	(-3.23)	(-5.84)	(-5.16)	(-0.35)	(-2.33)	(-5.04)
*PPE*	0.065	0.333***	0.011	0.021	0.009	0.044**
	(0.59)	(2.82)	(0.67)	(0.81)	(0.47)	(2.51)
*Board*	-0.092	-0.027	-0.003	-0.010	-0.014	-0.030**
	(-1.12)	(-0.28)	(-0.31)	(-0.94)	(-1.07)	(-2.37)
*lnAir*	-0.004	0.001	-0.001**	0.000	0.001*	-0.001
	(-0.73)	(0.24)	(-2.51)	(0.26)	(1.91)	(-1.26)
*lnRail*	0.151	0.147	0.022*	0.041***	0.013	0.003
	(1.09)	(1.22)	(1.73)	(2.72)	(0.58)	(0.18)
*lnRoad*	0.056	0.024**	0.001	0.001	0.003	0.015*
	(0.38)	(2.15)	(0.09)	(1.06)	(0.14)	(1.73)
*lnWater*	-0.034	-0.059**	-0.002	-0.001	-0.003	-0.002
	(-1.07)	(-2.21)	(-0.55)	(-1.21)	(-0.57)	(-1.23)
*Constant*	-1.473	-2.155	-0.153	-0.325**	3.947***	3.940***
	(-1.56)	(-1.39)	(-1.25)	(-2.31)	(6.50)	(6.95)
*Firm*	YES	YES	YES	YES	YES	YES
*Year*	YES	YES	YES	YES	YES	YES
*N*	7,029	4,403	7,029	4,403	7,029	4,403
*R* ^*2*^	0.284	0.340	0.171	0.158	0.180	0.109

## 6. Conclusion and inspiration

### 6.1. Analysis conclusion

This paper selects all A-share non-financial listed companies from 2008 to 2019, and uses the Difference-in-differences model to study the relationship between the opening of high-speed rail and corporate financing constraints, and draws the following conclusion: the opening of high-speed rail can alleviate the financing constraints of companies. Compared with state-controlled enterprises, the opening of high-speed rail has a more significant effect on alleviating the financing constraints of non-state-controlled enterprises. Compared with labor-intensive enterprises and capital-intensive enterprises, the opening of high-speed rail has a more significant effect on alleviating the financing constraints of technology-intensive enterprises. Compared with enterprises in coastal areas, the opening of high-speed rail has a more significant effect on alleviating financing constraints of inland enterprises. Further analysis of its influence mechanism shows that the opening of the high-speed railway can reduce the degree of information asymmetry and agency problems of enterprises, and then ease the financing constraints of enterprises.

### 6.2. Management enlightenment

#### 6.2.1. For government departments

Government departments should continue to optimize and develop the high-speed rail network, paying particular attention to the strategic deployment of opening high-speed rail in areas with underdeveloped economic systems. At present, the promotion effect of the opening of high-speed rail on the macro-economy has been confirmed. In the past two years, studies have continuously confirmed the micro-governance effect of the opening of high-speed rail on the capital market and enterprises. This paper further confirms that the opening of the high-speed rail has a significant alleviating effect on the financing constraints commonly faced by enterprises. Therefore, government departments should continue to promote the high-speed rail network layout and give full play to the positive effects of high-speed rail on business operation and management.

#### 6.2.2. For entrepreneurs

For enterprises, the direct enlightenment from this paper is that entrepreneurs should plan their offices to gather in cities where high-speed rail is opened. The problem of financing constraint generally exists in large and small enterprises in China, which has an important impact on the development of enterprises. The opening of high-speed rail can reduce the degree of information asymmetry of enterprises, improve information transparency, alleviate the agency problems of enterprises, and further ease the financing constraints of enterprises.

#### 6.2.3. For investors

For external investors, when making investment decisions, they can focus on companies that have opened high-speed rail in the city where the office is located. The opening of high-speed rail effectively reduces the space-time distance between investors and enterprises and promotes the transmission and exchange of information between various entities, improves information transparency, and alleviates financing constraints faced by enterprises, which has certain guiding significance for investors.
